# Twisted Right Fallopian Tube With a Large Fimbrial Cyst: A Diagnostic Challenge in Acute Pelvic Pain

**DOI:** 10.7759/cureus.94176

**Published:** 2025-10-09

**Authors:** Hani Bashir, Meldin Thomas, Magid Abubakar, Abdelaziz Satti

**Affiliations:** 1 Obstetrics and Gynaecology, The Coombe Hospital, Dublin, IRL; 2 Obstetrics and Gynaecology, Wexford General Hospital, Wexford, IRL

**Keywords:** acute abdomen, adnexal mass, fallopian tube torsion, fimbrial cyst, laparoscopy

## Abstract

Acute pelvic pain in women of reproductive age presents a diagnostic challenge, often mimicking surgical and gynecological emergencies such as appendicitis, ovarian torsion, or urolithiasis. Isolated torsion of the fallopian tube (IFTT) is a rare but important differential, with an incidence estimated at one in 1.5 million women. Fimbrial cysts are uncommon adnexal lesions that may predispose to torsion by increasing tubal mobility. We present a case of a 34-year-old woman, who presented to the emergency department with severe right iliac fossa pain, inconclusive preoperative imaging, and an intraoperative diagnosis of right fallopian tube torsion due to a large fimbrial cyst. Laparoscopic detorsion and cystectomy preserved tubal integrity. This case highlights the importance of early surgical intervention in suspected adnexal torsion to preserve fertility.

## Introduction

Isolated torsion of the fallopian tube (IFTT) is defined as the rotation of the fallopian tube on its longitudinal axis without involvement of the ipsilateral ovary [1]. Since first described by Bland-Sutton in 1890, fewer than 350 cases have been reported in the literature [2,3]. Predisposing factors include intrinsic abnormalities, such as hydrosalpinx, neoplasms, or congenital anomalies, and extrinsic factors, such as adhesions, pregnancy, or adnexal masses, including fimbrial and paratubal cysts [3]. Fimbrial cysts are benign lesions arising from paramesonephric or mesothelial remnants within the mesosalpinx [4]. While typically asymptomatic, they may facilitate torsion by increasing tubal mass and mobility. The condition is more often right-sided, possibly due to the protective effect of the sigmoid colon on the left and a diagnostic bias towards appendicitis in right lower quadrant pain [2]. Preoperative diagnosis is challenging, as clinical features are non-specific and imaging findings may overlap with other causes of pelvic pain. Ultrasound with Doppler is the preferred first-line imaging modality; the "whirlpool sign" representing a twisted vascular pedicle is suggestive but inconsistently visualized [4]. CT or MRI can help exclude other pathologies, but they are not definitive for IFTT. Early surgical exploration remains essential to confirm diagnosis and salvage the fallopian tube [1,3].

## Case presentation

A 34-year-old female, multiparous woman, para 3, presented to the emergency department with a 24-hour history of severe, continuous right iliac fossa pain radiating to the groin and flank. The pain was associated with nausea and multiple episodes of vomiting. She denied fever, urinary or bowel symptoms, and abnormal vaginal bleeding. She had a levonorgestrel-releasing intrauterine contraceptive device (IUCD, Mirena) in situ and a background of cervical intraepithelial neoplasia grade 3 (CIN 3), with a recent negative human papillomavirus (HPV) screen and normal cervical cytology. Surgical history included a laparoscopic appendectomy. Her family history was significant for breast cancer in first-degree relatives.

On examination, the patient appeared uncomfortable but was afebrile and hemodynamically stable. Abdominal examination revealed localized tenderness in the right iliac fossa without guarding or rebound tenderness. Pelvic speculum examination showed a healthy cervix with visible Mirena threads and no cervical motion tenderness. Laboratory investigations, including complete blood count, renal and liver function tests, and inflammatory markers, were all within normal limits. Urinalysis revealed trace protein and ketones, with a negative hCG result.

**Figure 1:**
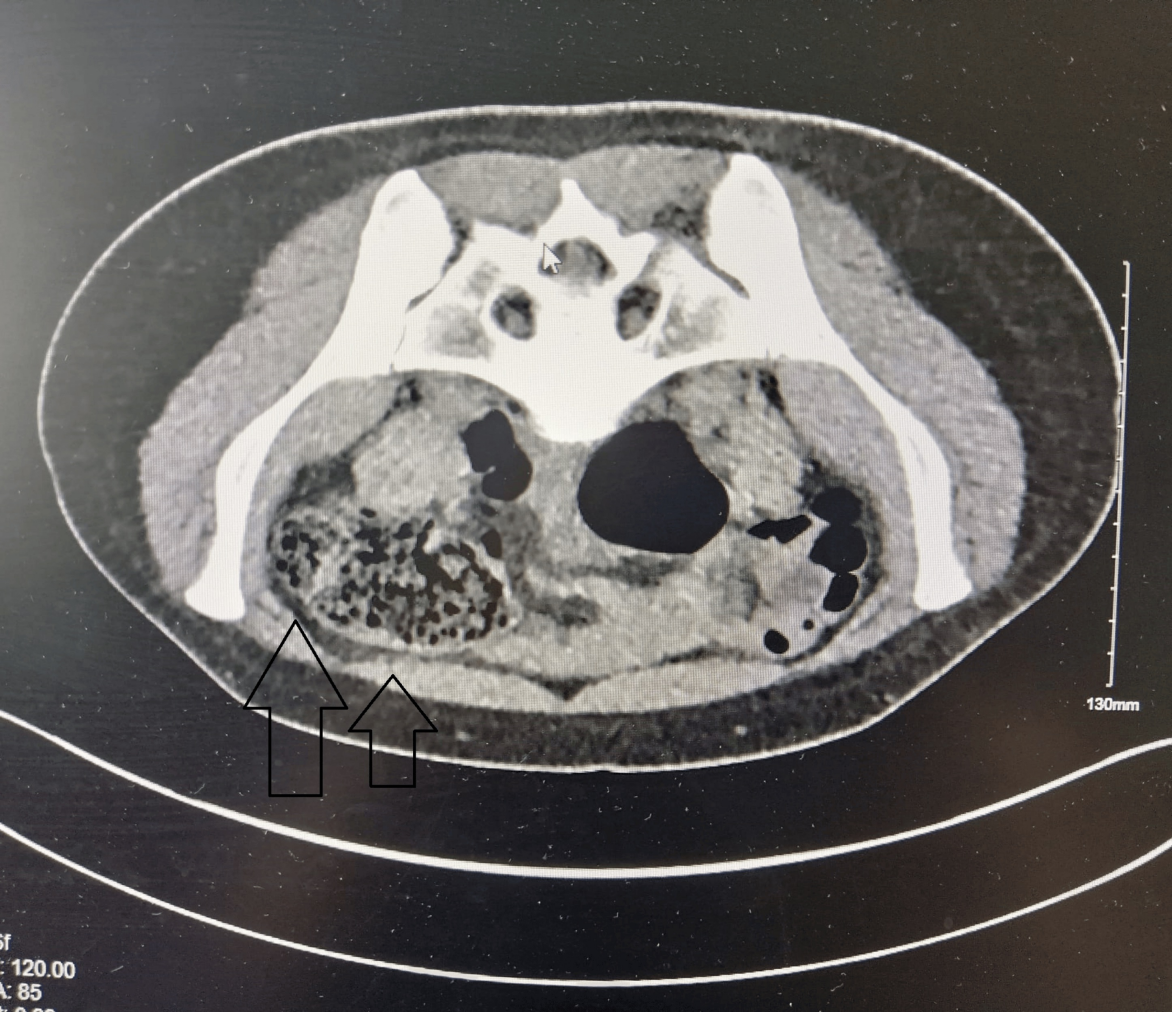
Axial CT-KUB (kidney, ureters, bladder) showing a large right adnexal cyst adjacent to the right ovary The image demonstrates a well-circumscribed, low-attenuation cystic lesion occupying the right adnexal region.

Given the severity of pain, clinical presentation, and concern for urolithiasis, the emergency team requested a CT scan of the kidneys, ureters, and bladder (CT-KUB) before a gynecology consultation. This revealed a large right adenexal cyst measuring 6 x 6.9 x 5.5 cm (TRV x AP x CC). Markedly smaller left adenexal cyst related to the ovary measuring 1.5 x 2 x 2.3 cm, likely a follicular cyst. An anteverted uterus with an IUCD was found, and there was no evidence of renal tract calculi and negative for free fluid and collection (Figure 1).

**Figure 2:**
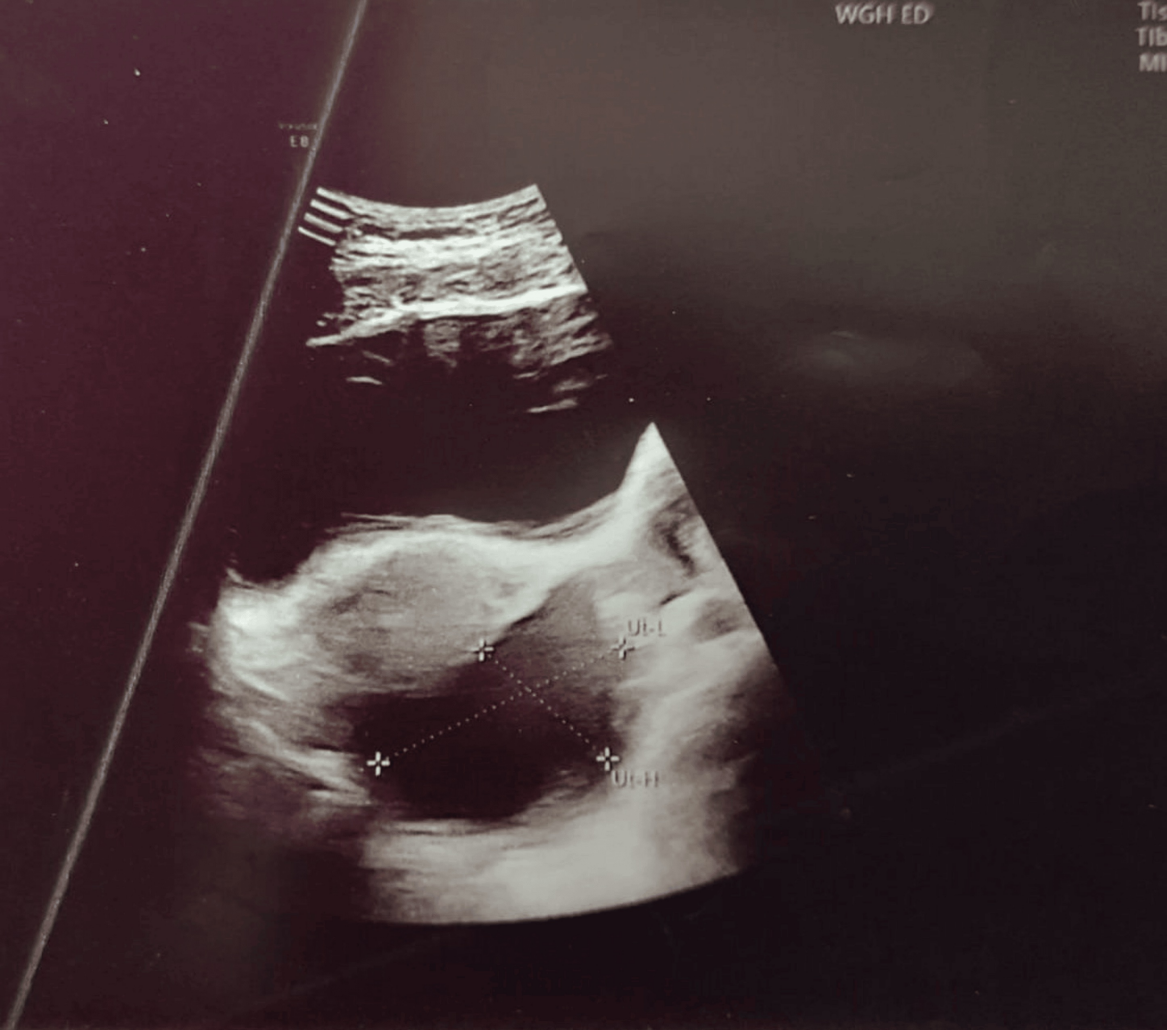
Transvaginal ultrasound (longitudinal view) showing an unilocular cystic lesion adjacent to the normal right ovary This image shows a cystic structure separate from the ovary, with a thin, smooth wall and no internal septations or solid components.

Subsequently, the gynecology team arranged a transvaginal pelvic ultrasound. This revealed a large cystic structure adjacent to the right ovary, which appeared morphologically normal with preserved blood flow on Doppler assessment (Figure 2).

**Figure 3:**
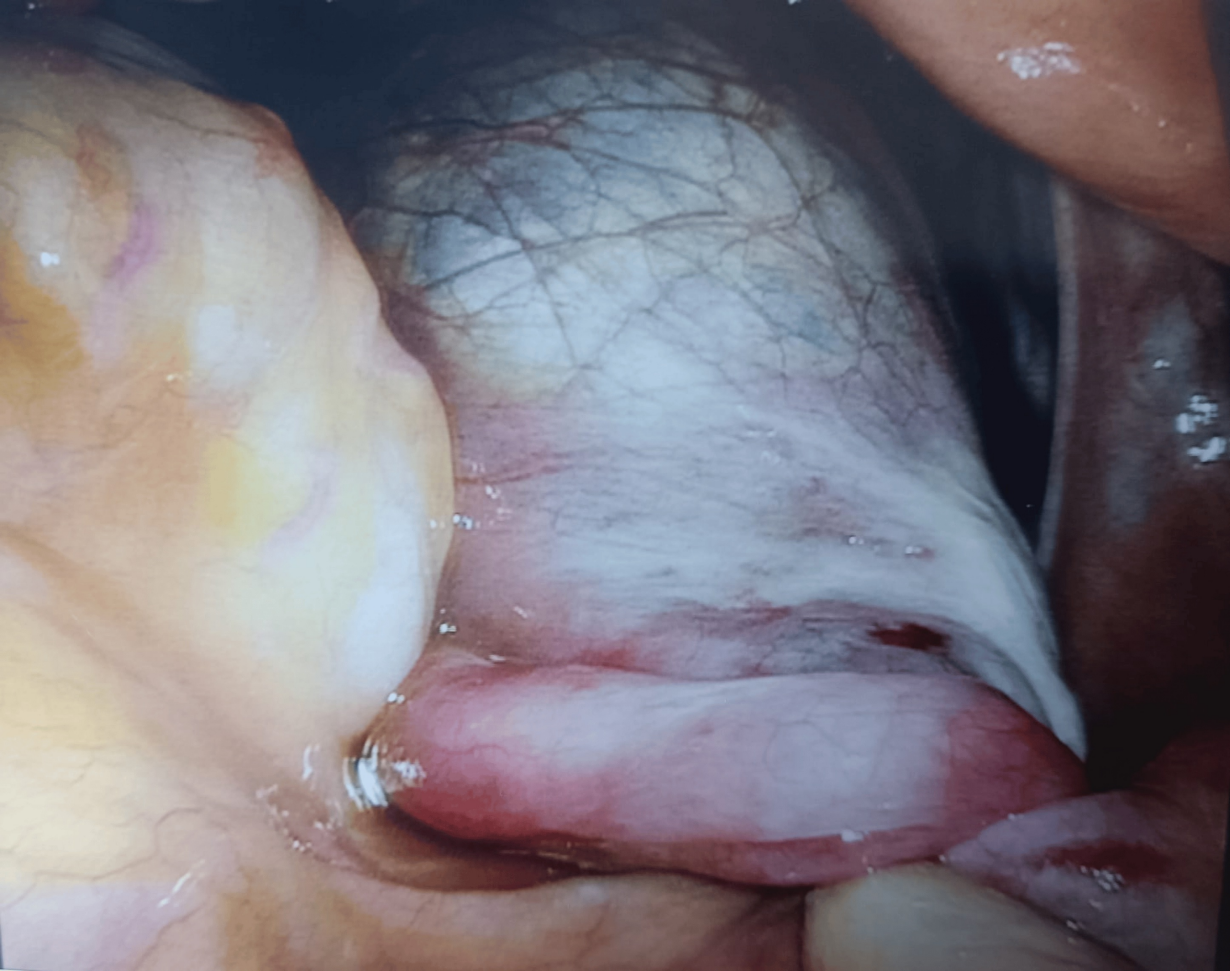
Laparoscopic image showing large fimbrial cyst with torsed right fallopian tube. The tube appears congested and mildly edematous, consistent with venous and lymphatic compromise.

**Figure 4:**
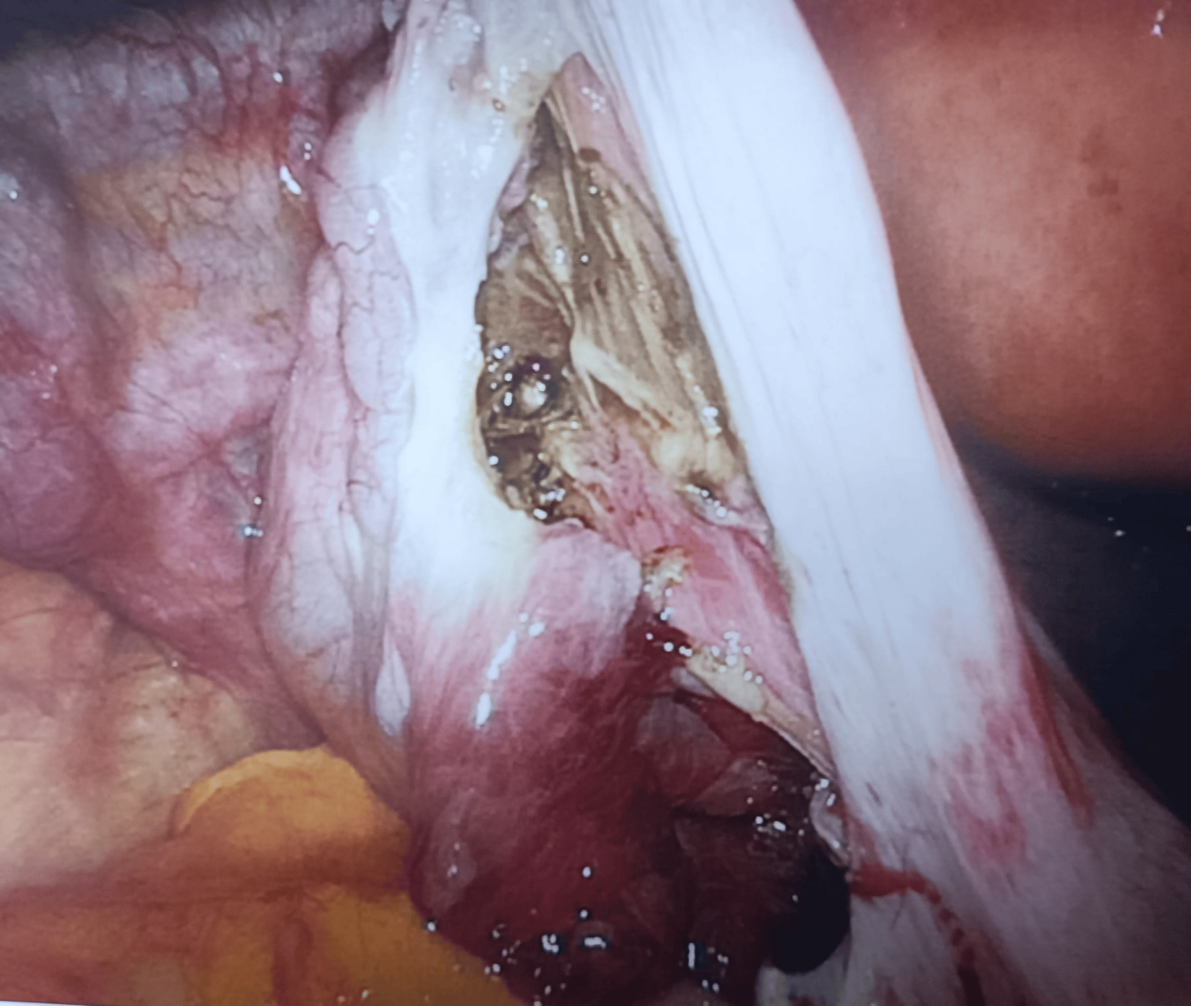
Detorsion of the right fallopian tube demonstrating viable tubal tissue

Due to ongoing pain despite analgesia and the presence of an adnexal cyst, diagnostic laparoscopy was performed. Intraoperative findings showed a normal uterus, both ovaries, and the left fallopian tube. A 7 × 7 cm right fimbrial cyst was identified with two complete twists of the right fallopian tube (Figure 3). Detorsion of the right tube and fimbrial cyst was drained, and the capsule was removed (cystectomy) (Figure 4). The intrauterine device was exchanged during the procedure. The excised specimen was sent for histopathology, which confirmed a benign serous cystadenoma with a markedly hemorrhagic and congested wall, suggestive of torsion. There was no evidence of malignancy.

The patient's postoperative course was uneventful, and she was discharged the next day with outpatient follow-up arranged. At the three-month follow-up, the patient remained asymptomatic, and a repeat pelvic ultrasound confirmed normal adnexal appearance with preserved right tubal structure. She was counselled about fertility preservation and recurrence risk and advised to report any recurrent pelvic pain promptly.

## Discussion

IFTT is a rare cause of pelvic pain, with an incidence estimated at approximately one in 1.5 million women [1], while fimbrial cysts constitute about 5-20% of adnexal masses [4,5]. It is characterized by the rotation of the fallopian tube on its axis without involvement of the ipsilateral ovary [2,3]. Torsion can occur due to intrinsic or extrinsic factors, including a long mesosalpinx, tubal cysts, and paratubal masses such as fimbrial cysts [4,5]. It is more commonly right-sided, likely due to the anatomical cushioning effect of the sigmoid colon on the left and the diagnostic bias toward appendicitis in right-sided pain [2,6]. Predisposing factors include intrinsic abnormalities such as hydrosalpinx, neoplasms, or congenital anomalies, and extrinsic causes including adhesions, pregnancy, or paratubal/fimbrial cysts [3-5]. Fimbrial cysts are benign paramesonephric or mesothelial remnants located in the mesosalpinx, and although usually asymptomatic, their presence may increase adnexal mobility and risk of torsion. 

The clinical presentation of IFTT is non-specific, often resembling appendicitis, ectopic pregnancy, or ovarian torsion [3,7]. Therefore, diagnosis is typically delayed or only made intraoperatively. While ultrasound remains the first-line imaging tool, findings may be inconclusive [6,8]. The “whirlpool sign” on Doppler imaging, representing a twisted vascular pedicle, may suggest torsion, but its sensitivity is variable [6]. CT and MRI can help rule out other causes and identify adnexal masses, but they are not definitive for IFTT [8].

Prompt surgical intervention is essential to avoid ischemic necrosis [9]. Several studies indicate that the risk of tubal necrosis increases significantly if surgery is delayed beyond 10-24 hours from symptom onset [9,10]. Conservative surgical management with detorsion and cystectomy is preferred in cases where the tube is viable, particularly in reproductive-aged women [9,10]. In our patient, a timely diagnostic laparoscopy allowed preservation of the adnexa and complete symptom resolution.

Emerging evidence supports a multidisciplinary approach involving emergency, surgical, and gynecologic teams in cases of unclear abdominal pain in women to optimize diagnostic accuracy and reduce unnecessary delays [8]. This collaborative model enhances patient outcomes and reduces morbidity. 

Limitations

This case report reflects an isolated clinical presentation and may not be generalizable. Selection and reporting biases are inherent to case studies. Long-term fertility outcomes remain observational. Nonetheless, it highlights the diagnostic value of multidisciplinary evaluation and timely intervention. 

## Conclusions

IFTT associated with a fimbrial cyst is a rare but important cause of acute pelvic pain in women of reproductive age. Due to its non-specific presentation and inconclusive imaging findings, diagnosis is often delayed, potentially leading to loss of reproductive anatomy. This case highlights the importance of considering fallopian tube torsion in the differential diagnosis of pelvic pain. Timely gynecologic consultation, appropriate imaging, and early laparoscopy are essential.

A multidisciplinary approach involving emergency medicine, radiology, surgery, and gynecology can enhance early recognition and management, preserve fertility, and improve clinical outcomes.
